# Genetic polymorphisms are associated with individual susceptibility to dexmedetomidine

**DOI:** 10.3389/fgene.2023.1187415

**Published:** 2023-08-24

**Authors:** Yuanyuan Ding, Aiqing Liu, Yafeng Wang, Shuai Zhao, Shiqian Huang, Hongyu Zhu, Lulin Ma, Linlin Han, Shaofang Shu, Lidong Zheng, Xiangdong Chen

**Affiliations:** ^1^ Department of Anesthesiology, Union Hospital, Tongji Medical College, Huazhong University of Science and Technology, Wuhan, China; ^2^ Department of Anesthesiology, Lu’an Hospital Affiliated to Anhui Medical University, Lu’an, China

**Keywords:** dexmedetomidine, pharmacogenomics, sedation, single nucleotide polymorphism, susceptibility

## Abstract

**Introduction:** Dexmedetomidine (DXM) is widely used as an adjuvant to anesthesia or a sedative medicine, and differences in individual sensitivity to the drug exist. This study aimed to investigate the effect of genetic polymorphisms on these differences.

**Methods:** A total of 112 patients undergoing hand surgery were recruited. DXM 0.5 μg/kg was administered within 10 min and then continuously injected (0.4 μg/kg/h). Narcotrend index, effective dose and onset time of sedation, MAP, and HR were measured. Forty-five single nucleotide polymorphisms (SNPs) were selected for genotype.

**Results:** We observed individual differences in the sedation and hemodynamics induced by DXM. *ABCG2* rs2231142, *CYP2D6* rs16947, *WBP2NL* rs5758550, *KATP* rs141294036, *KCNMB1* rs11739136, *KCNMA1* rs16934182, *ABCC9* rs11046209, *ADRA2A* rs1800544, and *ADRB2* rs1042713 were shown to cause statistically significant (*p* < 0.05) influence on the individual variation of DXM on sedation and hemodynamics. Moreover, the multiple linear regression analysis indicated sex, BMI, and *ADRA2A* rs1800544 are statistically related to the effective dose of DXM sedation.

**Discussion:** The evidence suggests that the nine SNPs involved in transport proteins, metabolic enzymes, and target proteins of DXM could explain the individual variability in the sedative and hemodynamic effects of DXM. Therefore, with SNP genotyping, these results could guide personalized medication and promote clinical and surgical management.

## 1 Introduction

Dexmedetomidine (DXM) has received much attention in recent years due to its sedative, hypnotic, analgesic, and anti-sympathetic properties, and it plays an indispensable role in the intensive care unit (ICU) and various surgeries (Yuki, 2021). The drug is a highly selective α2 adrenergic receptor agonist (Mei et al., 2021). With its hemodynamic stabilization, lack of respiratory depression, and other favorable physiological effects, DXM has gained increasing popularity in clinical and laboratory settings (Liu et al., 2021). Despite the increasing application in clinical anesthesia, much research has reported significant interindividual differences in DXM’s efficacy (Weerink et al., 2017). For example, Jakob and colleagues showed that a lack of efficacy could be found in approximately 1 in every 8 to 10 patients using DXM (Jakob et al., 2012); more seriously, Tellor and others proposed that its sedation failure rate was 21% in the ICU (Tellor et al., 2012). We also observed the individual variation in response to dexmedetomidine in clinic. Striking individual variance in its effect makes controlling the sedative extent in the clinic difficult. However, few studies have addressed this problem. Consequently, there is an urgent need to reveal the causes that may result in high interindividual variability of DXM.

Previous studies have demonstrated that clinical components, including age, sex, and body mass index (BMI), and genetic polymorphisms account for the difference in response to similar anesthetics (Xie et al., 2018). For instance, our previous studies observed that genetic variation in metabolic and functional pathways could result in individual variance in response to propofol, rocuronium, and sevoflurane (Zhong et al., 2017; Zhao et al., 2022; Zhu et al., 2022). DXM is mainly eliminated by the liver after intravenous infusion. Direct N-glucuronidation by uridine 5′-diphospho-glucuronosyltransferase (UGT2B10 and UGT1A4) and hydroxylation mediated by cytochrome P450 (CYP) enzymes (CYP2A6) constitute the main metabolic pathways (Ber et al., 2020). Sedative and hypnotic effects resembling natural sleep by DXM are thought to be mediated through the activation of central pre- and post-synaptic α2 adrenergic receptors in the locus coeruleus (Choi et al., 2021). Additionally, DXM regulates vasoconstriction or vasodilation by binding with α2 receptors in several structures (De Cassai et al., 2021). α2 adrenergic receptors are specifically encoded by ADRA2A gene. Similarly, β2 adrenergic receptor, which is encoded by ADRB2 and located in cardiovascular system widely, also plays a major role in hemodynamics (Dai et al., 2021). Apart from these, many Sodium, potassium or calcium channels are involved in vasoconstriction or vasodilation (Kawano et al., 2012; Scruggs et al., 2020). Therefore, these genes are assumed to be linked with pharmacodynamics response to DXM. Together, a bunch of genetic polymorphisms in drug pharmacokinetics and pharmacodynamics were hypothesized to influence the individual variance in DXM strongly.

We designed this project to describe and examine whether genetic polymorphisms in metabolic enzymes and receptors could explain the personal discrepancy in the effect of DXM.

## 2 Materials and methods

### 2.1 Ethics

The investigations were carried out following the rules of the Declaration of Helsinki of 1975, revised in 2013. Ethical approval for this study (approval number, 2019-S1205) was provided by the Institutional Ethics Committee of Tongji Medical College, Huazhong University of Science and Technology, Wuhan, China (Chairperson Prof Hui Chen) on 20 November 2019. Written informed consent was obtained from all patients.

### 2.2 Study population

We enrolled 112 patients undergoing hand surgery with brachial plexus nerve block at Union Hospital, Tongji Medical College, Huazhong University of Science and Technology from December 2019 to August 2020. They were not genetically related to each other. We adopted the following inclusion criteria: 1) written informed consent; 2) scheduled for elective hand surgery; 3) aged 18–80 years; 4) American Society of Anesthesiologists (ASA) Physical Status I or II; 5) a BMI <30; and (6) no history of surgery and drug addiction. The exclusion criteria included 1) allergy to any of the drugs used in the study; 2) liver or renal function dysfunction; 3) sinus bradycardia or moderate to severe atrioventricular block; 4) treated with vasoactive or other drugs during the perioperative period; and 5) pregnancy or lactation.

### 2.3 Assessment of sedation by DXM

Given the Narcotrend monitoring (MT MonitorTechnik, Bad Bramstedt, Germany) and Observer’s Assessment of Alertness/Sedation (OAA/S) Scale, which have been used as validated tools to assess the depth of sedation (Kreuer et al., 2003; Schrier et al., 2017), the sedation condition was evaluated by a trained assessor with the Narcotrend index (NI) and modified OAA/S scores (Pastis et al., 2019).

### 2.4 Study protocol

All patients routinely received standard monitoring of electrocardiogram (ECG), noninvasive blood pressure (NIBP), peripheral pulse oximetry, and Narcotrend brain monitoring. The mean arterial pressure (MAP), heart rate (HR), pulse oxygen saturation (SpO_2_), and NI were recorded continuously. Simultaneously, intravenous access was established. Subsequently, ultrasound-guided intermuscular groove brachial plexus block and axillary brachial plexus block were performed by the same experienced anesthesiologist. In our implementation, the method we used was partly based on Senel’s study (Senel et al., 2014) with the following modifications: 1) none of the patients received preoperative medication before the block; 2) the local anesthetic solution consisted of 20 mL 1% lidocaine and 20 mL 0.5% ropivacaine, were 20 mL local anesthetic was injected into the intermuscular groove and axillary groove, respectively; and 3) nerve stimulators were not used. When the sensory and motor functions were blocked completely, DXM was pumped within 10 min at a loading dose of 0.5 μg/kg and then continuously pumped at 0.4 μg/kg/h during the surgery. We documented the MAP, HR, and NI before the pump (T0) and 5 (T1), 10 (T2), 15 (T3), and 20 (T4) minutes after the pump started. Another researcher assessed the OAA/S scores at 5-min intervals up to 20 min. OAA/S score of 2 was believed to the ideal sedation state in the current study (Laporte et al., 2011). Once the patient did not arrive at ideal sedation within the first 20 min, we kept evaluating until it did. The total amount of DXM and total injection time were recorded as OAA/S scores of 4, 3, 2, 1, and 0 (Table 1). Supplemental oxygen by face mask was delivered continuously. Any patient treated with vasoactive or other drugs during the perioperative period was excluded from the analysis. Above that, all the patients were just given the local anesthetic solution and DXM.

**TABLE 1:** Observer’s assessment of alertness/sedation (OAA/S) Scale.

Score	Description
5	Responds readily to name spoken in normal tone
4	Lethargic response to name spoken in normal tone
3	Responds only after name is called loudly and/or repeatedly
2	Responds only after mild prodding or shaking
1	Responds only after painful trapezius squeeze
0	Does not respond to painful trapezius squeeze

### 2.5 Outcome measures

The primary outcome was the relationship between the single nucleotide polymorphism (SNP) and DXM sedation. The indices of the sedative effect included OAA/S scores and NI values. And the secondary outcome was the relationship between the SNP and the hemodynamic consequence of DXM. The hemodynamic indices included MAP, HR, and percent changes in MAP and HR. The percent change in MAP was calculated by the formula [(actual measured MAP value - baseline MAP value)/baseline MAP value] × 100%; the percent change in HR was calculated by the formula [(actual measured HR value - baseline HR value)/baseline HR value] × 100%. Considering the small number of patients with the minor allele for some SNPs, all patients were separated into two groups depending on the genotyping results: 1) homozygous for the major allele and 2) either heterozygous or homozygous for the minor allele.

The total amount of DXM and total injection time at an OAA/S score of 2 were chosen to determine the susceptibility to DXM as the “effective dose” and “onset time”, respectively. On the other hand, the differences in MAP and HR at the four time points mentioned above (T1 to T4) were used to detect cardiovascular susceptibility to DXM.

### 2.6 DNA sample collection and SNP genotyping

The genetic sample was obtained from the dorsal hand vein of each patient and collected in an EDTA-containing blood tube at the end of the operation. Then, they were instantly stored frozen at −80°C, and we extracted the blood later using standard phenol–chloroform procedures. Thus, the DNA was isolated from the frozen blood. Sequenom Mass ARRAY SNP genotyping systems were then used to determine genotypes, which were based on detection through MALDI-TOF MS (Sequenom Inc., San Diego, CA, United States).

### 2.7 Gene and SNP selection

The selection of these SNPs was based on our knowledge of metabolic pathways, drug distribution, drug excretion, targets, and the mechanism of DXM action after an extensive literature study. And these SNPs were confirmed to be relatively common in the Asian population and were therefore considered to have potential clinical significance. Eventually, from essential proteins to important SNPs, 45 SNPs of 28 genes were chosen as candidates (Table 2). Among the total candidates, three genes are known to be responsible for the pharmacokinetics of DXM (*CYP2A6*, *UGT1A4*, *UGT2B10*) (Holliday et al., 2014; Ber et al., 2020), and ten analyzed genes were speculated to be associated with DXM pharmacokinetics (*ABCG2*, *ABCB1*, *ABCC2*, *ABCC3*, *SLCO1B1*, *SLC22A1*, *CYP1A2*, *CYP2C19*, *CYP2D6*, and *WBP2NL*). Additionally, the ADRA2A gene has been demonstrated to participate in the primary mechanism of DXM action (Zhu et al., 2019; Choi et al., 2021). The remaining fourteen genes were hypothesized to be involved in the pharmacodynamics of DXM (*SLC31A1*, *KATP*, *KCNJ11*, *KCNMB1*, *KCNMA1*, *KCNN4*, *CACNA1D*, *CACNA1C*, *ABCC9*, *ADRB2*, *COMT*, *GNB3*, *PRKCB*, and *PRKCH*).

**TABLE 2:** List of the candidate genes and polymorphisms.

Gene	SNP ID	Alleles	MAF	HWE *p*-value^a^	Characteristics
Transport proteins					
*ABCG2*	rs2231142	*G>T*	0.25	0.20	non-synonymous
*ABCB1*	rs1045642	*G>A*	0.36	0.17	non-synonymous
*ABCC2*	rs717620	*C>T*	0.21	0.78	5′ UTR
*ABCC3*	rs4793665	*T>C*	0.15	1.00	2 KB upstream
*SLCO1B1*	rs4149056	*T>C*	0.14	0.83	non-synonymous
*SLC22A1*	rs4646277	*C>T*	0	—	non-synonymous
	rs2282143	*C>T*	0.15	0.99	non-synonymous
Metabolic enzymes					
*UGT1A4*	rs2011425	*A>C*	0.21	0.18	non-synonymous
	rs2011404	*C>T*	0.02	0.98	non-synonymous
	rs3732217	*G>A*	0.22	0.71	synonymous
*UGT2B10*	rs835309	*G>T*	0.49	<0.001	intron
*CYP2A6*	rs28399468	*C>A*	0.03	0.96	non-synonymous
	rs5031016	*A>G*	0	—	non-synonymous
	rs28399433	*T>G*	0.16	0.17	2 KB upstream
	rs56113850	*T>C*	0.32	<0.001	intron
*CYP1A2*	rs762551	*A>C*	0.40	0.46	intron
*CYP2C19*	rs4244285	*G>A*	0.33	0.20	synonymous
	rs12248560	*C>T*	0.01	0.99	2 KB upstream
*CYP2D6*	rs16947	*G>A*	0.15	0.26	non-synonymous
	rs3892097	*G>A*	0.01	0.99	splice acceptor
	rs1065852	*C>T*	0.38	<0.001	non-synonymous
	rs28371725	*G>A*	0.04	0.93	intron
*WBP2NL*	rs5758550	*A>G*	0.09	0.62	intron
Target proteins					
*SLC31A1*	rs10981694	*T>G*	0.27	0.40	intron
*KATP*	rs141294036	*C>T*	0.05	0.86	intron
*KCNJ11*	rs5215	*T>C*	0.33	0.07	non-synonymous
*KCNMB1*	rs11739136	*C>T*	0.08	0.70	non-synonymous
*KCNMA1*	rs16934182	*G>A*	0.02	0.94	intron
	rs1131824	*G>A*	0.24	0.70	synonymous
*KCNN4*	rs2306799	*G>A*	0.29	0.48	intron
*CACNA1D*	rs312481	*G>A*	0.11	0.47	intron
	rs3774426	*C>T*	0.13	0.92	intron
*CACNA1C*	rs16929277	*C>G*	0.02	0.99	intron
*ABCC9*	rs11046209	*A>T*	0.11	0.47	intron
*ADRA2A*	rs13306146	*A>G*	0.36	0.22	3′ UTR
	rs1800544	*G>C*	0.30	0.76	2 KB upstream
	rs553668	*A>G*	0.46	0.21	3′ UTR
	rs775887911	*C>T*	0.01	1.00	non-synonymous
	rs11195419	*C>A*	0.14	0.77	3′ UTR
*ADRB2*	rs1042718	*C>A*	0.32	0.32	non-synonymous
	rs1042713	*A>G*	0.42	0.66	non-synonymous
*COMT*	rs4680	*G>A*	0.28	0.89	non-synonymous
*GNB3*	rs5443	*T>C*	0.48	0.90	synonymous
*PRKCB*	rs9922316	*G>T*	0.03	0.96	intron
*PRKCH*	rs2230500	*G>A*	0	—	non-synonymous

^a^ HWE *p,*-value <0.05 indicated deviation from equilibrium.

SNP, single nucleotide polymorphism; MAF, minor allele frequency; HWE, Hardy-Weinberg equilibrium.

### 2.8 Statistical analysis

Statistical analyses were performed using SPSS 23.0 or GraphPad Prism 8.0 software. All data are expressed as the means ± standard deviations (SD). We applied Akaike information criterion to select the best model of inheritance and inheritance modeling suggested a dominant model for all SNPs, that is all patients were divided into two groups based on genotyping results, the homozygous for the major allele and the group of heterozygous and homozygous for the minor allele. We adopted Shapiro–Wilk test to examine the normal distribution of data, and the homogeneity of variance was assessed by *F* test. The differences in clinical characteristics among different time points were compared by one-way ANOVA. Independent-sample two-tailed *t*-test or Mann–Whitney *U* test was utilized to analyze the differences in NI values, percent changes of MAP or HR, onset time, effective dose and clinical characteristics between the homozygous for the major allele and the group of heterozygous and homozygous for the minor allele. The chi-squared test was selected to evaluate the candidate SNPs’ Hardy-Weinberg equilibrium (HWE). Finally, a multiple linear regression analysis was added to measure the effect of significant SNPs and clinical factors (age, sex, and BMI) on the effective dose. Commonly, *p* < 0.05 was utilized to consider the deviation from equilibrium or statistical significance.

## 3 Results

### 3.1 General information

As 20 patients did not meet the inclusion criteria (2 patients were overweight, 15 did not have phenotypic data, and 3 were treated with vasoactive drugs due to excessively high preoperative BP), 92 of the 112 patients were recruited for the follow-up analysis in this study. The demographics of all study patients were referred to in Table 3.

**TABLE 3:** Demographic information of the study patients.

Demographics	Results
Age (years)	41.88 ± 14.40
Male/female	63/29
BMI	23.21 ± 3.04
Race	Han Chinese
Concomitant medication	None

Data are expressed as mean ± SD, or n.

BMI, body mass index; SD, standard deviation.

### 3.2 Clinical characteristics and individual susceptibility to DXM

**FIGURE 1:**
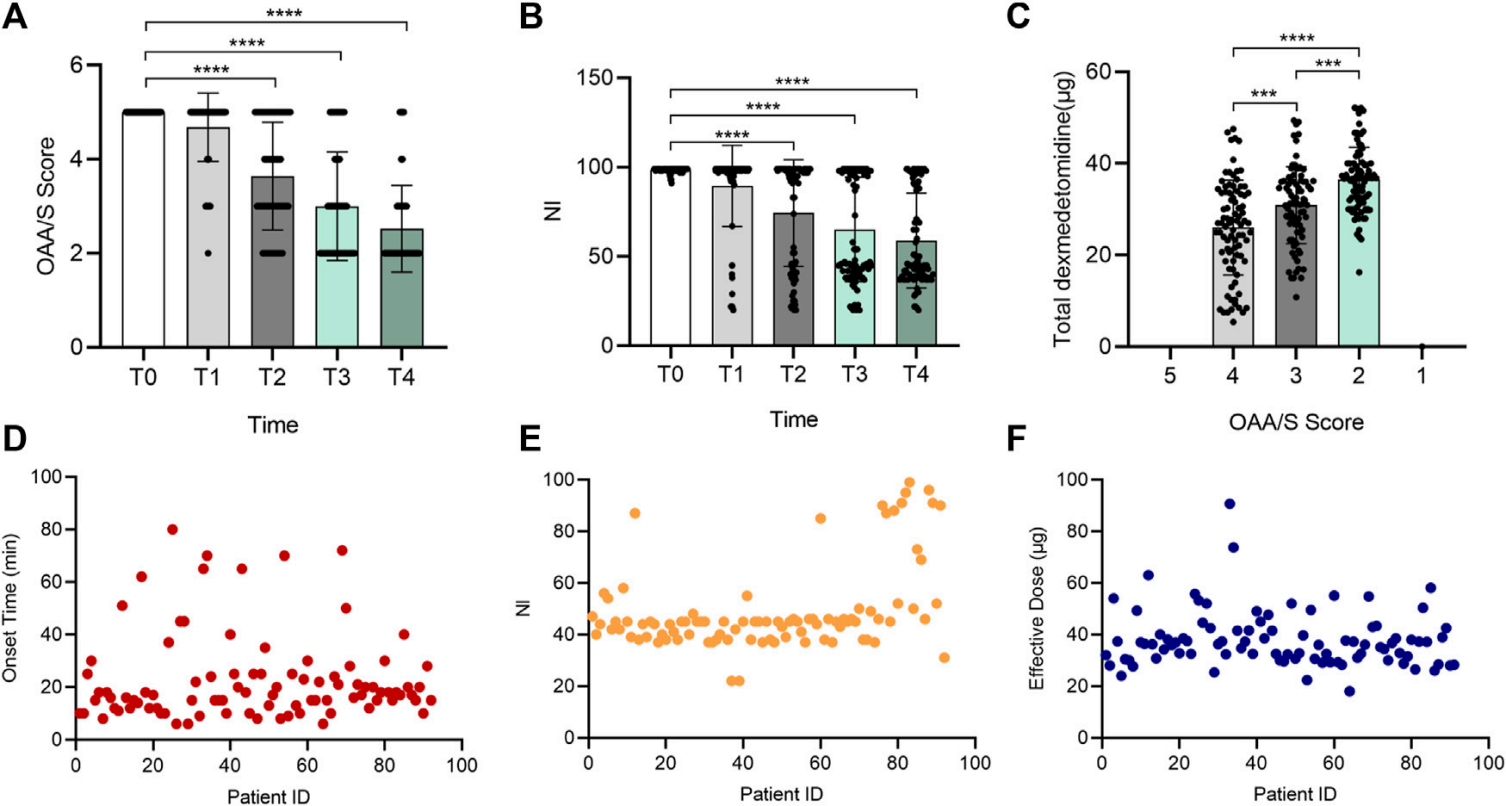
Individual variation in the sedative effect of DXM. Bar chart **(A)** of OAA/S scores at the 5 time points (T0–T4), bar chart **(B)** of NI values at the 5 time points (T0–T4), bar chart **(C)** of total DXM dose at the 5 time points (T0–T4), and **(D–F)** the distribution of all participants’ onset time **(D)**, NI values **(E)**, and effective dose **(F)** at the OAA/S score of 2, respectively. In **(D–F)**, the horizontal axis represents the patients included in the analysis, and they were not ordered to show how discrete the data is. The vertical axis represents each subject’s corresponding onset time, NI value, or effective dose. Data in **(A–C)** are expressed as means ± SD, data in **(D–F)** are expressed as raw data. Variance between different groups was analyzed by one-way ANOVA. ***p < 0.001, ****p < 0.0001. *n* = 92. DXM, dexmedetomidine; OAA/S, Observer’s Assessment of Alertness/Sedation; NI, Narcotrend index.

As shown in Figure 1, the sedative effect induced by DXM was time- and dose-dependent. With the continuous injection of DXM, the OAA/S score decreased from 5 to 4.68 ± 0.73, 3.64 ± 1.14, 3 ± 1.16, and 2.52 ± 0.92 at T1, T2, T3, and T4, respectively (Figure 1A). Simultaneously, NI decreased from 98.51 ± 1.35 to 89.48 ± 22.73, 74.32 ± 29.87, 65.08 ± 29.28, and 58.89 ± 26.52 (Figure 1B). The total amount of DXM required for OAA/S scores of 4, 3, and 2 was 26.01 ± 10.34, 30.89 ± 8.38, and 36.43 ± 7.11 µg, respectively (Figure 1C). The onset time, NI and effective dose of each patient were displayed in Figures 1D–F, respectively. Figure 1D shows a marked individual variation in the dose-response under DXM sedation. First, 13.33-fold variability existed between the fastest and slowest onset times, which ranged from 6 to 80 min and averaged 22.72 ± 16.45 min. According to Figure 1E, NI value at OAA/S of 2 mainly fell into 30–50 in 71 patients despite the maximum and minimum, which were 22 and 99, differing by 4.5 times. The variable effective dose also showed a similar result, ranging from 18 to 90.67 µg when the OAA/S was 2 (Figure 1F).

**FIGURE 2:**
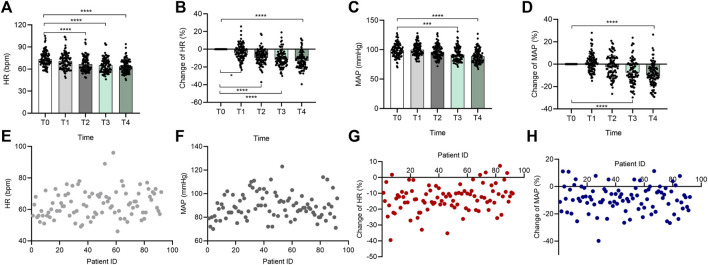
Individual variation in the cardiovascular responses to DXM. Bar chart **(A)** of HR at the 5 time points (T0–T4), bar chart **(B)** of percent change of HR at the 5 time points (T0–T4), bar chart **(C)** of MAP at the 5 time points (T0–T4), bar chart **(D)** of percent change of MAP at the 5 time points (T0–T4), **(E–H)** the distribution of all participants’ HR **(E)**, MAP **(F)**, percent change of HR **(G)**, and percent change of MAP **(H)** at the OAA/S score of 2. In **(E–H)**, the horizontal axis also represents the patients included in the analysis, and they were not ordered to show how discrete the data is. The vertical axis represents each subject’s corresponding HR, MAP, percent change of HR, or percent change of MAP. Data in **(A–D)** are expressed as means ± SD, data in **(E–H)** are expressed as raw data. Variance between different groups was analyzed by one-way ANOVA. *p < 0.05, ***p < 0.001, ****p < 0.0001. *n* = 92. DXM, dexmedetomidine; HR, heart rate; MAP, mean arterial pressure.

DXM also has a statistically significant effect on systemic hemodynamics. HR and MAP both depicted a time- and concentration-dependent change tendency (Figure 2). With a greater degree of sedation, as shown in Figures 2A,B, the HR declined from 73.42 ± 10.32 to 70.54 ± 10.88 bpm, to 66.60 ± 10.32, to 65.09 ± 9.77 bpm, and to 63.70 ± 8.98 bpm from T1 to T4, and the percent alterations in HR were −3.72% ± 8.82%, −9.09% ± 8.31%, −11.03% ± 8.97%, and −12.78% ± 9.18% at the above time points. A similar phenomenon was observed in the MAP (Figures 2C,D), which was diminished from 98.88 ± 11.43 (T0) mmHg to 96.30 ± 11.01 (T2) mmHg, 91.54 ± 11.72 (T3) mmHg, and 88.90 ± 11.18 (T4) mmHg, except for a modest increase found at the start (elevated to 99.95 ± 10.59 (T1). Thus, the MAP was changed by 1.50% ± 7.95%, −2.08% ± 9.85%, −6.95% ± 10.42%, and −9.63% ± 9.99% from T1 to T4, respectively. Further analysis showed that at an OAA/S of 2, there were large individual differences in patients’ HR and MAP in response to DXM (Figures 2E–H). The data in Figures 2E,F suggested the HR ranged from 46 to 96 bpm, and the MAP ranged from 70 to 123 mmHg. The percent changes in HR and MAP ranged from −39.53% to 7.35% and from −39.84% to 11.36%, respectively (Figures 2G,H).

### 3.3 Genotyping results

The genotype distributions of the 45 SNPs and the minor allele frequency (MAF) of each SNP are shown in Table 2. No mutations in *CYP2A6* rs5031016, *SLC22A1* rs4646277, or *PRKCH* rs2230500 were found in these patients. All the patients carried the major alleles. Except for *UGT2B10* rs835309, *CYP2A6* rs56113850, and *CYP2D6* rs1065852, allele frequency distributions of the 22 polymorphic sites were in HWE (*p* > 0.05).

### 3.4 Univariate analysis results

The NI, onset time, effective dose, and percent changes in HR and MAP at OAA/S scores of 2 were selected to assess the susceptibility to DXM. As indicated in Table 4, *ABCG2* rs2231142, *CYP2D6* rs16947, *WBP2NL* rs5758550, *KATP* rs141294036, *KCNMB1* rs11739136, *KCNMA1* rs16934182, *ABCC9* rs11046209, *ADRA2A* rs1800544, and *ADRB2* rs1042713 were found to be linked with sensitivity to sedative or hemodynamic effect of DXM.

**TABLE 4:** SNPs with detected statistically significant differences in clinical indexes.

Genotype	Patients	NI	MAP (%)	HR (%)	Onset time (min)	Effective dose (ug)
*ABCG2* rs2231142						
*GG*	55 (60)	48.15 ± 18.00	−8.09 ± 8.63	−13.78 ± 8.35	19.02 ± 12.72	35.73 ± 6.90
*GT + TT*	37 (40)	50.65 ± 15.45*	−10.49 ± 10.80	−12.46 ± 8.17	28.22 ± 19.72*	41.21 ± 14.61
*CYP2D6* rs16947						
*GG*	65 (71)	49.05 ± 16.42	−8.54 ± 10.39	−12.98 ± 8.36	22.58 ± 15.57	39.22 ± 11.76
*GA + AA*	27 (29)	49.41 ± 18.59	−10.30 ± 7.28	−13.90 ± 8.11	23.04 ± 18.70	34.84 ± 8.09*
*WBP2NL* rs5758550						
*AA*	75 (82)	47.15 ± 15.82	−8.19 ± 9.96	−12.75 ± 8.51	22.28 ± 15.81	38.51 ± 11.17
*AG + GG*	17 (18)	58.00 ± 19.51*	−12.85 ± 6.68*	−15.46 ± 6.82	24.65 ± 19.41	35.37 ± 9.86
*KATP* rs141294036						
*CC*	82 (89)	49.83 ± 17.43	−9.80 ± 9.40	−13.70 ± 8.10	22.65 ± 16.62	37.50 ± 9.64
*CT + TT*	10 (11)	43.60 ± 12.01	−2.94 ± 9.33*	−9.57 ± 9.04	23.30 ± 15.76	41.48 ± 19.01
*KCNMB1* rs11739136						
*CC*	77 (84)	49.65 ± 17.33	−8.61 ± 9.74	−13.98 ± 8.11	23.91 ± 16.47	37.82 ± 10.79
*CT + TT*	15 (16)	46.60 ± 15.34	−11.34 ± 8.65	−9.51 ± 8.22	16.60 ± 15.42*	38.52 ± 12.15
*KCNMA1* rs16934182						
*GG*	89 (97)	49.37 ± 17.22	−8.58 ± 9.36	−13.27 ± 8.38	23.06 ± 16.61	38.23 ± 11.02
*GA + AA*	3 3)	42.67 ± 2.52	−23.07 ± 4.41*	−12.73 ± 3.56	12.67 ± 2.52	29.16 ± 1.38*
*ABCC9* rs11046209						
*AA*	71 (77)	46.56 ± 14.75	−9.75 ± 9.34	−13.62 ± 7.81	21.80 ± 15.61	37.87 ± 10.97
*AT + TT*	21 (23)	57.90 ± 21.12*	−6.69 ± 10.25	−12.01 ± 9.73	25.81 ± 19.11	38.13 ± 11.17
*ADRA2A* rs1800544						
*GG*	43 (47)	46.63 ± 13.62	−7.90 ± 8.93	−13.07 ± 8.08	19.23 ± 10.43	35.49 ± 7.46
*GC + CC*	49 (53)	51.37 ± 19.33	−10.06 ± 10.10	−13.41 ± 8.49	25.78 ± 19.93	40.08 ± 12.99*
*ADRB2* rs1042713						
*AA*	29 (32)	49.34 ± 17.40	−8.24 ± 9.79	−14.25 ± 7.80	18.55 ± 7.65	35.80 ± 8.30
*AG + GG*	63 (68)	49.06 ± 16.93	−9.43 ± 9.54	−12.79 ± 8.48	24.63 ± 18.94*	38.91 ± 11.91

Data are expressed as n (%) or mean ± SD.

Independent-sample two-tailed *t*-test or Mann–Whitney *U* test was utilized to analyze the differences in NI, values, percent changes of MAP or HR, onset time, and effective dose between the homozygous for the major allele and the group of heterozygous and homozygous for the minor allele.

*p < 0.05 (homozygous carriers of the major allele vs. carriers of the minor allele) was considered statistically significant.

SNP, single nucleotide polymorphism; NI, narcotrend index; MAP, mean arterial pressure; HR, heart rate; SD, standard deviation.

#### 3.4.1 *ABCG2* rs2231142

In terms of sedative effect, carriers of the minor allele (GT/TT) showed a higher NI value (50.65 ± 15.45 vs. 48.15 ± 18.00, *p* = 0.044) than homozygotes for the major allele (GG). A similar result was found for the indicator of onset time (28.22 ± 19.72 vs. 19.02 ± 12.72, *p* = 0.047). These results suggested that homozygosity for the major allele G) of *ABCG2* rs2231142 may be linked to increased sensitivity to DXM.

No statistically significant differences were observed in the impact of the gene mutation on hemodynamic parameters.

#### 3.4.2 *CYP2D6* rs16947

In our study, homozygous carriers of the major allele (GG) took larger effective doses than those either heterozygous (GA) or homozygous for the minor allele (AA) (39.22 ± 11.76 vs. 34.84 ± 8.09, *p* = 0.02). The results revealed that carriers of the minor allele A) required less DXM to induce sedation.

#### 3.4.3 *WBP2NL* rs5758550

On average, homozygous carriers of the major allele (AA) showed significantly lower NI values than those either heterozygous (AG) or homozygous for the minor allele (GG) (47.15 ± 15.82 vs. 58.00 ± 19.51, *p* = 0.008). Based on the results, homozygosity for the major allele A) of *WBP2NL* rs5758550 may be related to higher sensitivity to the DXM’s sedation.

In addition, mutation of the gene rs5758550 also played a vital role in the effect of DXM on blood pressure. In our study, homozygosity for the major allele significantly changed less in MAP during DXM sedation period than either heterozygosity or homozygosity for the minor allele (−8.19 ± 9.96 vs. −12.85 ± 6.68, *p* = 0.025). Thus, heterozygosity or homozygosity for the gene’s minor allele G) may cause a higher susceptibility to the cardiovascular impact of DXM.

#### 3.4.4 *KATP* rs141294036

In our study, homozygous carriers of the major allele (CC) produced a bigger absolute value for the percent changes in MAP than those either heterozygous (CT) or homozygous for the minor allele (TT) (−9.80 ± 9.40 vs. −2.94 ± 9.33, *p* = 0.032). Homozygosity for the major allele C) of *KATP* rs141294036 can be inferred to be associated with greater susceptibility to the cardiovascular impact of DXM.

#### 3.4.5 *KCNMB1* rs11739136

In our study, carriers of the minor allele (CT/TT) needed a shorter onset time than homozygous carriers of the major allele (CC) (16.60 ± 15.42 vs. 23.91 ± 16.47, *p* = 0.009). Therefore, heterozygosity or homozygosity for the minor allele T) of *KCNMB1* rs11739136 resulted in more sensitizing to DXM sedation.

#### 3.4.6 *KCNMA1* rs16934182

In our study, carriers of the minor allele (GA/AA) required less DXM to reach the ideal sedation status than homozygosity for the major allele (GG) (29.16 ± 1.38 vs. 38.23 ± 11.02, *p* = 0.034). Heterozygosity or homozygosity for the minor allele A) of *KCNMA1* rs16934182 was a factor in more sensitizing to DXM sedation.

Concerning the hemodynamic effect, we discovered the patients with GA/AA had a greater percent change in MAP than those with GG (−23.07 ± 4.41 vs. −8.58 ± 9.36, *p* = 0.009). Therefore, heterozygosity or homozygosity for A allele of *KCNMA1* rs16934182 was more sensitive to the cardiovascular effect of DXM.

#### 3.4.7 *ABCC9* rs11046209

In this research, homozygous carriers of the major allele (AA) presented a significantly lower NI than those either heterozygous (AT) or homozygous for the minor allele (TT) (46.56 ± 14.75 vs. 57.90 ± 21.12, *p* = 0.002). The evidence points to the likelihood that homozygosity for the A allele of *ABCC9* rs11046209 may increase sensitivity to DXM.

#### 3.4.8 *ADRA2A* rs1800544

In this research, homozygous carriers of the major allele (GG) required less DXM than carriers of the minor allele (GC/CC) (35.49 ± 7.46 vs. 40.08 ± 12.99, *p* = 0.038). Thus, homozygosity for the major allele G) of *ADRA2A* rs1800544 may be involved in a higher sensitivity to DXM.

#### 3.4.9 *ADRB2* rs1042713

In our study, homozygosity for the A allele (AA) was more quickly to arrive at the desired sedation status than carriers of the G allele (AG/GG) (18.55 ± 7.65 vs. 24.63 ± 18.94, *p* = 0.031). This means that homozygosity for the major allele A) of *ADRB2* rs1042713 was associated with a higher susceptibility to DXM’s efficacy.

To exclude the effects of sex, age, BMI, and basal NI in our pharmacogenetic analysis, we further examined the correlation between clinical features and genotypes of significant SNPs. Except for the BMI composition in *CYP2D6* rs16947 (*p* = 0.012) and basal NI in *ADRA2A* rs1800544 (*p* = 0.021), no statistically significant difference was observed in these clinical parameters between patients who were homozygous for the major allele and patients who were either heterozygous or homozygous for the minor allele for each of the tested SNPs.

### 3.5 Multiple linear regression analysis of the relationship between multiple factors and susceptibility to DXM sedation

Considering that the major pharmacological effect of DXM is sedation and the effective dose represents a comprehensive measure of the sedative effect observed in each participant after administration, we applied multiple linear regression analysis to further test the effect of independent variables on the susceptibility to DXM sedation (effective dose). The significant SNPs (*CYP2D6* rs16947, *KCNMA1* rs16934182, and *ADRA2A* rs1800544) that are associated with individual variation in response to DXM sedation, and clinical traits (age, sex, and BMI) were selected as independent variables. The effective dose of DXM was selected as the dependent variable. As illustrated in Table 5, sex, BMI, and *ADRA2A* rs1800544 were statistically significant associated with the individual sensitivity to DXM sedation (F = 6.685, *R* ^2^ = 0.321, *p* < 0.001). Additionally, the significant clinical variables (sex and BMI) and one significant SNP *ADRA2A* rs1800544 in the model accounted for 27.3% of the variability in DXM dosage for sedation (adjusted *R* ^2^ = 0.273).

**TABLE 5:** Multiple linear regression analysis of clinical variables and SNPs related to the sensitivity to the sedative effect of dexmedetomidine (effective dose).

Independent variables	Standardized coefficients	*t*	*p*
Clinical variables			
Age	0.068	0.735	0.464
Sex	−0.271	−2.881	0.005*
BMI	0.366	3.803	<0.0001*

SNPs			
*CYP2D6* rs16947	−0.073	−0.778	0.439
*KCNMA1* rs16934182	−0.114	−1.224	0.224
*ADRA2A* rs1800544	0.206	2.295	0.024*
Model fit: *R* = 0.566, *R* ^*2*^ = 0.321, adjusted *R* ^*2*^ = 0.273, DW = 1.826, *F* = 6.685, *p* < 0.001			

SNP, single nucleotide polymorphism; DW: Durbin-Watson test.

## 4 Discussion

In this study, we systemically investigated the genetic polymorphism roles of metabolic enzymes, target sites of channels or receptors, and transporters of DXM in individual differences in response to DXM. Our results showed obvious individual differences in sedative and hemodynamic effects of DXM: different patients required different onset times and effective doses, and different hemodynamic responses occurred at the same sedation level. Several gene polymorphisms, including *ABCG2* rs2231142, *CYP2D6* rs16947, *WBP2NL* rs5758550, *KATP* rs141294036, *KCNMB1* rs11739136, *KCNMA1* rs16934182, *ABCC9* rs11046209, *ADRA2A* rs1800544, and *ADRB2* rs1042713, were shown to be partly responsible for the susceptibility to DXM sedation or hemodynamic response. Furthermore, a multiple linear regression analysis was conducted and revealed that sex, BMI, and *ADRA2A* rs1800544 were linked with sensitivity to the sedative effect of DXM. This phenomenon reflects James’ study, which also found that weight was a significant predictor of DXM clearance (James et al., 2021). Among the above statistically significant genes, *ABCG2*, *CYP2D6* and *ADRA2A*, *ADRB2* are the most common genes responsible for the pharmacokinetics and pharmacodynamics of DXM, respectively. Therefore, we would discuss these genes in detail.

### 4.1 *ABCG2* gene

ABCG2, the ATP-binding cassette, subfamily G, isoform 2 protein, encoded by the *ABCG2* gene, is a key member of the ABC transporter superfamily, removing substrates from the cell in an energy-dependent manner (Kukal et al., 2021). ABCG2 is expressed in the gastrointestinal system, kidney, liver and blood–brain barrier and protects organisms against xenobiotic exposure (Zhang et al., 2018). Numerous studies have demonstrated that *ABCG2* SNPs affect the pharmacokinetics of many therapeutic drugs, such as topotecan, sunitinib, and risperidone (Stacy et al., 2013; Werbrouck et al., 2019; Ganoci et al., 2021). Our study indicated that the rs2231142 polymorphism in the *ABCG2* gene could influence the patients’ NI values at an OAA/S score of 2 and their onset times. As reported by previous studies (Bauer et al., 2016; Al-Majdoub et al., 2019), patients with GT or TT alleles in *ABCG2* rs2231142 seem to have a shorter onset time and deeper sedation, because TT is associated with reduced function. The possible reason is mutant allele increased the rate of ABCG2 degradation in the endoplasmic reticulum *via* ubiquitination and proteosomal proteolysis (Furukawa et al., 2009). Contrary to expectations, our result found that patients with GT or TT alleles were more awake (higher NI values) and had a longer onset time. A possible explanation for this might be that *ABCG2* rs2231142GT or TT allele tends to reduce more MAP or tends to have more pronounced vasodilation. After that, the blood concentration of DXM is reduced, and patients with GT or TT alleles are more awake (higher NI score) and have a longer onset time. Another possible explanation is that previous studies were conducted among white or black people. They did not include Asians. To our knowledge, this is the first study to investigate the correlation between the *ABCG2* SNP and sensitivity to DXM. In other words, our current findings expand on prior work.

### 4.2 *CYP2D6* gene and rs5758550

Many cytochrome P450 enzymes are encoded in the human genome. They are involved in a wide range of metabolic pathways, including the biotransformation of endogenous and exogenous molecules, dietary components, and drugs (Saravanakumar et al., 2019). Among the CYP superfamily members, CYP2D6 is one of the most important and extensively studied drug-metabolizing enzymes (Owen et al., 2009). Previous studies have described that highly genetic polymorphisms in CYP2D6, influencing the enzymatic function of CYP2D6, were associated with the efficacy of some drugs, including some antidepressants, antineoplastic agents, and analgesics (Nofziger et al., 2020; Taylor et al., 2020). Moreover, clinical guidelines have adopted CYP2D6 metabolism status into therapeutic recommendations for CYP2D6-substrate drugs (Taylor et al., 2020). Rs16947, one of the common SNPs in *CYP2D6*, is known to reduce CYP2D6 mRNA expression two-fold by affecting exon 6 splicing (Wang et al., 2014). Apart from itself, rs5758550, located 115 kbp downstream of the *CYP2D6* promoter, resides within a pivotal enhancer region directing CYP2D6 expression. The “enhancer” SNP was suggested to affect overall CYP2D6 mRNA expression (Wang et al., 2015; Dinh et al., 2021). These findings provide support for our current results.

Based on our results, the rs16947 polymorphism in the *CYP2D6* gene was related to the less effective dose of DXM, perhaps because of decreased CYP2D6 expression, thus, prolonging the duration of drug action. And rs5758550 significantly elevated the patients’ NI values and percent change in MAP. The data indicated that the two genes had a different impact on DXM’s efficacy, which contradicts the phenomenon above (Dinh et al., 2021). However, the two SNPs related to the *CYP2D6* yield alteration in the same direction in every clinical index (Table 4). Considering those similarities and differences, more research is needed to explore the role of rs5758550. Similar to *ABCG2*, this is the first report of an association between *CYP2D6* genetic polymorphisms, rs5758550, and DXM clinical efficacy.

### 4.3 *ADRA2A* gene

Among three highly homologous subtypes (α2A-, α2B-, and α2C-adrenergic receptors), DXM produces sedation, analgesia, and sympathetic nervous system inhibition mainly by activating the central pre- or post-synaptic α2A-adrenergic receptor (ADRA2A), which is encoded by the *ADRA2A* gene (Zhu et al., 2019). Much effort has been made to explore the linkage between several genetic polymorphisms in the *ADRA2A* gene and susceptibility to DXM (Fu et al., 2020). The present study also demonstrated that patients with the mutation (G>C) in *ADRA2A* rs1800544 need more DXM to reach the ideal sedative extent. Our result agrees with Yagar’s conclusion that patients with the mutant allele in *ADRA2A* rs1800544 showed higher bispectral index and Ramsay sedation scores, signifying a longer period before falling asleep (Yağar et al., 2011). Small’s work consistently pointed out that the mutant of rs1800544 decreased ADRA2A expression (Small et al., 2006). Additionally, Zhu and colleagues reported that the rs1800544 polymorphism could manipulate heart rate changes in patients after rocuronium infusion (Zhu et al., 2019), which expands our work. Collectively, *ADRA2A* rs1800544 mutation plays an indispensable role in differences in individual sensitivity to DXM, even in other efficacy of other drugs.

### 4.4 *ADRB2* gene

The *ADRB2* gene encodes the β2-adrenoreceptor, which plays a vital role in regulating the cardiovascular system (Dai et al., 2021). Several SNPs in the *ADRB2* gene could impact cardiovascular activity, thereby altering the risk of hypertension (El-Menyar et al., 2015; Huang et al., 2018). Nielsen and colleagues found that carriers of the minor allele (G) showed a tendency toward vasodilation and high cardiac output during anesthesia. The effect could result in receiving more ephedrine (Nielsen et al., 2016). In addition, our research proved that homozygosity or heterozygosity of G allele in *ADRB2* rs1042713 would demand a longer onset time. One possible explanation for this discrepancy is that the rs1042713 mutation would dilate blood vessels and ultimately require infusion of more DXM to gain the same effect-chamber concentration. Moreover, recent studies have identified the SNP to alter the expression and conformation of β2-adrenoreceptor (Xie et al., 2019). Thus, when determining the amount of DXM to produce the appropriate sedation status, the hemodynamic effect should be taken into consideration.

There are a few limitations in our study. First, the study sample size may limit our ability to identify more significant associations. And we are prepared to perform further research to expand the cohort. In the current study, multiple testing correction would likely be ineffective in capturing potentially relevant SNPs associated with the phenotype. Considering that our study was aimed to identify more potential genetic markers associated with the variability in individual response to DXM, we did not do adjustments like FDR or Bonferoni. Second, we did not measure the plasma concentrations of DXM, and we used the total infusion dose instead. As a result, the pharmacokinetic analysis of this study was inadequate. We would take the question into account in further research and then provide a better distinction between pharmacokinetic and pharmacodynamic variations.

## 5 Conclusion

In summary, our present study primarily reported that *ABCG2* rs2231142, *CYP2D6* rs16947, *WBP2NL* rs5758550, *KATP* rs141294036, *KCNMB1* rs11739136, *KCNMA1* rs16934182, *ABCC9* rs11046209, *ADRA2A* rs1800544, and *ADRB2* rs1042713 were significantly associated with susceptibility to the sedative or hemodynamic effect of DXM. This work provides compelling evidence for the influence of genetic polymorphisms on the effect of DXM. It could help to predict individual variability in oversedation or insufficient sedation and hypotensive blood pressure in DXM sedation. Eventually, this could lead to the clinical optimization of DXM. Furthermore, the approach applied in the research has potential in areas such as investigating individual differences in the effects of other anesthetics.

## Data Availability

The raw data supporting the conclusion of this article will be made available by the authors, without undue reservation.
